# New Approaches in the Development of a Vaccine for Mucosal Candidiasis: Progress and Challenges

**DOI:** 10.3389/fmicb.2012.00294

**Published:** 2012-08-13

**Authors:** Anna Vecchiarelli, Eva Pericolini, Elena Gabrielli, Donatella Pietrella

**Affiliations:** ^1^Microbiology Section, Department of Experimental Medicine and Biochemical Sciences, University of PerugiaPerugia, Italy

**Keywords:** mucosal candidiasis, *C. albicans*, vaccine, fungal infections, candidiasis

## Abstract

The commensal fungus *Candida albicans* causes mucosal candidiasis in the rapidly expanding number of immunocompromised patients. Mucosal candidiasis includes oropharyngeal, esophageal, gastrointestinal, and vaginal infections. Vulvovaginal candidiasis (VVC) and antimycotic-refractory recurrent VVC is a frequent problem in healthy childbearing women. Both these mucosal infections can affect the quality of life and finding new therapeutical and preventive approaches is a challenge. A vaccine against candidal infections would be a new important tool to prevent and/or cure mucosal candidiasis and would be of benefit to many patients. Several *Candida* antigens have been proposed as vaccine candidates including cell wall components and virulence factors. Here we discuss the recent progress and problems associated with vaccination against mucosal candidiasis.

## Introduction

*Candida albicans* is a dimorphic fungus that colonizes different areas of the body from the gastrointestinal tract to oral and vaginal mucosa. It is usually a commensal microorganism but in immunocompromised or otherwise debilitated hosts it can cause disseminated and mucosal candidiasis. Systemic candidiasis is the fourth most common hospital-acquired infection in the USA (Pfaller et al., 1998a,b). The associated mortality depends on host conditions, and ranges between 30 and 50% (Pfaller et al., 1998b; Kibbler et al., 2003). Invasive fungal infections are common and severe in patients with hematologic malignancies, leading to particularly high mortality. Somewhat different from systemic infections, mucosal infections are common not only in immunocompromised patients but also in apparently normal subjects (Kirkpatrick, 2001). Their most common sites are the oral cavity, and the gastrointestinal and vaginal tracts. Oral thrush is one of the most frequent clinical forms of mucocutaneous candidiasis. It occurs at all ages with aggressive symptoms especially in infants and elderly people (Lopez-Martinez, 2010).

Vulvovaginal candidiasis (VVC) is a common distressing infection that affects up to 75% of childbearing women worldwide at least once in their life. Up to 7% of these women suffer from frustrating recurrent infection (RVVC) defined as at least three or four episodes of acute VVC in 1 year (Fidel, 2007; Sobel, 2007). Two forms of RVVC have been described: idiopathic (Id) primary RVVC with unknown predisposing factors, and secondary RVVC which occurs under predisposing conditions such as antibiotic treatment, oral contraceptive use, or diabetes mellitus (Fidel, 2004). These infections affect the quality of life, and require frequent antimycotic treatment enhancing the risk of acquiring drug resistance (Cassone, 2007).

Overall, the growing impact of fungal diseases has resulted in renewed interest in new approaches for improving their control. Among these new approaches, the generation of fungal vaccines is recognized as a priority. A mucosal vaccine would improve the quality of life of a long list of target populations of women suffering from RVVC. The aim of this review is to discuss the recent progress and problems associated with the development of a protective mucosal vaccine against candidiasis.

## Innate Immune Response at Mucosal Surfaces

The immunopathology associated with mucosal candidiasis is still not completely understood. The presence of *C. albicans* as a commensal on the mucosal surface does not go unnoticed or tolerated by the host – humoral and cellular factors of the innate and acquired immune response play an important role in limiting the growth of the fungus and neutralizing the activity of the virulence factors (Cassone et al., 2007). Indeed the transition from asymptomatic colonization to symptomatic infection occurs when the local defenses are impaired and when the fungus becomes more aggressive causing epithelial damage and inflammation through the production of virulence factors. In addition, alteration of the normal microbiota plays an important role.

The mucosal microbiota is acquired by colonization when passing through the birth canal, and soon after birth. The commensals establish their residence in mucosal niches where they replicate and play a crucial role in the development of the immune system. The immune response to the microbiota at the mucosal surface helps to maintain barriers to potentially harmful microorganisms (Pirofski and Casadevall, 2012). *C. albicans* as a commensal microorganism is part of normal microbiome in specific niches such as the oral cavity, vagina, and gut in at least 50% of healthy individuals. However it can cause pathology following alterations of the local environment and/or impairment of the immune response. To persist within the human host and cause disease at the mucosal surfaces, *C. albicans* has acquired several distinct factors enabling these microorganisms to adhere to and invade host cells.

Epithelial cells (ECs) lining the mucosa play an essential role in the defense against *C. albicans*. Indeed at the mucosal surface ECs sense the presence of *Candida* and elaborate a number of mechanisms to tolerate its presence and limit adherence, penetration, and damage. It is clear that ECs recognize the fungus and are capable of distinguishing its different forms of growth (Moyes et al., 2010), however the relative role of pattern recognition receptors (PRRs) in ECs is still largely unclear, despite the intensive research in PRR-mediated interaction of *C. albicans* with myeloid cells (Netea et al., 2006, 2008). The recognition of *C. albicans* by these cells has largely been elucidated and the major PRRs and their putative ligands derived from *C. albicans* have been extensively studied (Roeder et al., 2004a,b; Netea et al., 2006). It is known that ECs express PRRs: toll-like receptors (TLRs), dectin-1, and galectins (Backhed and Hornef, 2003; Hornef and Bogdan, 2005; Weindl et al., 2007). TLR2 and TLR5 are expressed at particularly high levels and these receptors have been associated with biological functions of ECs such as growth, survival, and repair (Rhee et al., 2005; Shaykhiev et al., 2008).

## *Candida* Recognition at the Mucosal Surface

Compelling evidence shows that *C. albicans* recognition by TLR4 on monocytes/macrophages results in release of proinflammatory cytokines such as IL-1 and TNF-α (Netea et al., 2006) and also the stimulation via TLR2 and FcγRII leads to TNF-α production (Netea et al., 2008). TLR4, which is highly expressed on myeloid cells and plays a critical role in driving immune response against *C. albicans* (Netea et al., 2006), is poorly expressed in ECs at the oral mucosal surface (Backhed and Hornef, 2003), and it seems that TLR4 does not have a role in cytokine induction in response to *Candida* (Li and Dongari-Bagtzoglou, 2009) and to other TLR4 ligands such as LPS (Naglik and Moyes, 2011).

At present, which fungal cell wall structures are recognized in oral ECs is also unknown, however a recent study has established that human oral ECs have a direct antifungal activity demonstrating the important role of these cells in mucosal protection (Weindl et al., 2007). Indeed, the oral epithelium is able to induce various defense effector molecules (Diamond et al., 2008) and to orchestrate an immune response to activate immune cells in the submucosal layers to clear the invading pathogens (Cutler and Jotwani, 2006). It has also been reported that after fungi recognition, infected ECs are activated and they produce proinflammatory cytokines and chemokines including IL-8 which induces neutrophil recruitment (Weindl et al., 2007; Moyes and Naglik, 2011) into the mucosa surface. This could be considered a mechanism of immune surveillance (Moyes and Naglik, 2011). However, despite the beneficial role of these cells in protection against systemic candidiasis, PMNs seem to exert no protective role in vaginal candidiasis. Neutrophils are abundantly recovered during vaginal candidiasis, but their presence was not associated to reduction of fungal burden (Fidel et al., 1999). On the contrary, it has been hypothesized that ECs have high sensitivity to *Candida* and secrete S100 alarmin resulting in a vigorous PMN migration which results in an inflammatory response and symptoms of infection (Yano et al., 2010). In agreement with this, a live challenge model in humans recently revealed that symptomatic vaginitis is caused by an aggressive innate response (neutrophil infiltrates and elevated fungal burden in the vaginal lumen); the protection has been associated to a non-inflammatory innate response (Fidel, 2007). Therefore the capacity of ECs to recruit and activate neutrophils might be a double-edged sword, with both favorable and unfavorable consequences depending on the specific niche.

There is no doubt, therefore, that many aspects of EC interaction with *C. albicans*, including the recognition of specific antigens and regulation of the complicated network of regulatory activities that characterize commensalism and infection in different niches, remain to be studied.

## T Cell Response in Mucosal Candidiasis

The role of T cells in mucosal candidiasis has only been partially elucidated, and the attempt to integrate the defense response to systemic and mucosal candidiasis remains inconclusive (Ashman et al., 2011). There is a wealth of studies reporting that Th1-type immunity is dependent on the presence of IL-12 in the milieu (Trinchieri, 2003) and compelling evidence indicates that the Th1 response is protective against mucosal candidiasis. The beneficial role of the Th1 response in oral candidiasis in particular has been underlined (Schaller et al., 2004). In agreement with these findings is the marked susceptibility of HIV-infected subjects to oral candidiasis when CD4^+^ T cells are depleted, suggesting a role for both Th1 and Th17 cell functional CD4 subsets. In addition, depletion of IL-12 resulted in acute susceptibility to oral infection in a mouse experimental model (Farah et al., 2002; Fidel, 2006; Zakikhany et al., 2007). In a report by Conti et al. (2009) susceptibility to oropharyngeal candidiasis (OPC) in mice with impaired Th1 and/or Th17 responses was compared. In this study fungal infections of the tongue were less severe in mice lacking IL-12p35 than in mice lacking IL-23p19, suggesting that Th17 responses play a central role in control of infection. However, the fungal burden in both IL-12p35- and IL-23p19-deficient mice was substantially higher than in controls, evidencing that the absence of either Th1 or Th17 cells impaired the ability of the mice to limit fungal growth (Pirofski and Casadevall, 2009). As well as driving innate immunity and neutrophil responses, Th17 cells have also been shown to drive antibody responses at mucosal surfaces, in particular secretory IgA (Moyes and Naglik, 2011).

## Humoral Response in Mucosal Candidiasis

For many years antibodies have been considered irrelevant in the host defense against invasive candidiasis, but evidence for this mode of protection has been mounting over the last two decades. Indeed a number of antibodies or their engineered derivatives directed against *C. albicans* cell wall compounds have been shown to confer protection (Cutler et al., 2007, 2011; Cassone and Rappuoli, 2010; Xin and Cutler, 2011). The discovery of numerous antigens on the fungal cell wall that elicit protective antibody responses raises the possibility of vaccines designed with multiple antigens, and/or passive therapies that combine antibodies with different specificities (Casadevall et al., 2004; Casadevall and Pirofski, 2007).

Protective antibodies have been reported for *C. albicans* cell wall polysaccharides, proteins, and peptides (De Bernardis et al., 1997; Matthews et al., 2003; Cutler, 2005; Yang et al., 2005; Xin et al., 2008). As a prevention strategy, protection against disease may be actively or passively acquired by vaccination and the administration of preformed monoclonal antibodies (Xin and Cutler, 2011).

In early studies protection against vaginal candidiasis was associated with the presence of protective antibodies against *Candida* constituents in the vaginal fluids and increased number of activated lymphocytes in the vaginal mucosa (De Bernardis et al., 1997; de Bernardis et al., 2000). More recently the same group demonstrated that vaginal B cells play an important role in protection against vaginal candidiasis suggesting that the contribution of T cells is predominantly that of helping B cells in protective antibody production (De Bernardis et al., 2010). Recently, treatment with synthetic peptides whose sequences are identical to fragments from the constant region of different classes of antibodies has been shown to provide protection against experimental vaginal candidiasis (Polonelli et al., 2012), and a protective effect in vaginal candidiasis was also noticed after passive administration of anti-β-glucan mAbs (Torosantucci et al., 2009). Unfortunately, the role of Abs has rarely been investigated in other mucosal infections such as oral candidiasis.

## Anti-*Candida* Vaccines and Protection Mechanisms

Recent papers support the idea that it may be possible to develop an antifungal vaccine that grants protection against multiple fungal pathogens (Cassone and Rappuoli, 2010; Stuehler et al., 2011; Wuthrich et al., 2011). Some studies have focused on cell-mediated immunity as the mechanism of protection (Spellberg et al., 2008), which is in accordance with the literature (Xin et al., 2008), whereas others (Cutler et al., 2007; Karwa and Wargo, 2009; Torosantucci et al., 2009) have determined that antibodies specific to certain *Candida* antigens are protective. Because of the variegate nature of *Candida* virulence and participation of different host responses in protection, it may well be possible that both humoral and cellular responses play a role in vaccination, providing the necessary collaboration to grant protection.

Several papers suggest that the induction of strong cellular immunity plays a paramount role in protection against candidiasis. One approach for stimulating cellular immunity was priming *ex vivo* dendritic cells (DCs) with fungal cells and re-administering the loaded DCs to the host (Roy and Klein, 2012). Less recent papers showed that DCs transfected with fungal RNA adoptively transferred into otherwise susceptible recipients, did in fact confer protection against *C. albicans* by inducing a protective Th1 response (Bozza et al., 2004; Perruccio et al., 2004). Recently a novel antigenic peptide, derived from a cell wall-associated adhesion and recognized by a major population of all *Candida*-specific Th cells isolated from infected mice, was isolated and sequenced from infected DCs. This peptide contributes to fungal pathogenicity and it is conserved in many clinically important *Candida* species such as *C. dubliniensis* and *C. glabrata*. Of note is the fact that human Th cells also responded to stimulation with the peptide. Furthermore when this peptide is used in combination with an adjuvant inducing IL-17A secretion, it acts as an efficient vaccine by protecting mice from fatal candidiasis (Bar et al., 2012).

The fungal-specific antibody-mediated protection may occur not only through classical immunological responses such as phagocytosis and killing and complement activation, but also through direct antibody actions on fungal cells, and even though antibody protection is considered specific many fungal antigens may be used for vaccination and to obtain therapeutic immunoglobulins (Casadevall and Pirofski, 2012). The novel fully synthetic β-(Man)3-Fba glycopeptide vaccine that represents two epitopes of *C. albicans* cell surface has been shown to be protective in an experimental model of systemic candidiasis (Xin et al., 2008). The protection was afforded with a strong antibody response that was achieved by using a DC/Complete Freund Adjuvant (CFA) combination (Xin et al., 2008). Indeed the antigen-pulsed DC proved to be a powerful means to induce a potent immune response that was not achieved by using small carbohydrate and peptide antigens of *C. albicans*. More recently, by coupling β-(Man)3-Fba glycopeptide to tetanus toxoid to render this vaccine entirely compatible with human use, a high degree of antibody-mediated protection was observed (Xin et al., 2012). These vaccine formulations also proved protective in mucosal models of infection (Xin et al., 2012). Another important effort to induce protection against *C. albicans* was made by Li et al. (2011) who showed that treatment with recombinant enolase, an important glycolytic enzyme located on the cell wall of *C. albicans*, conferred a protective effect against systemic challenge evaluated by fungal burdens in target organs, titers of specific antibodies to enolase, and levels of Th1/2 cytokines in serum.

## Vaccine Candidates for Mucosal Candidiasis

Strategies to elaborate a vaccine for mucosal candidiasis should take into account what type of immune response is protective under natural conditions, but should not necessarily be limited to mimicking the natural history of mucosal infection and protection. Hence, several studies have focused on different strategies to induce protection against vaginal candidiasis. In particular, it has been reported that vaccination with the recombinant N terminus of the candidal adhesin rAls3p-N protects mice against disseminated and oropharyngeal and vaginal candidiasis by a cell-mediated immune response (Th1/Th17). Antibodies have also been generated but their titers did not correlate with protection. The rAls3p-N vaccine is a promising new vaccine candidate for further exploration to prevent systemic and mucosal candidal infections (Spellberg et al., 2006). It has recently completed a Phase 1 clinical trial and proved to be safe and immunogenic. In particular, this research group reported that vaginal and systemic protective responses could be achieved by vaccination with rAls3-N which stimulates Th1/Th17 lymphocytes to produce high levels of IFN-γ and IL-17A, as well as the chemokines KC and MIP-1. These cytokines enhance the capacity of phagocytes to kill the pathogen. This vaccine protects immunocompetent mice from both vaginal candidiasis and lethal disseminated candidiasis and it also significantly reduces oral fungal burden in the corticosteroid-treated mouse model of OPC. Human trials with the rAls3-N vaccine are in final preparation (Liu and Filler, 2011). An additional approach to achieve protection against mucosal infection was immunization with *C. albicans* dsDNA which induces host resistance in newborn mice against gastrointestinal *C. albicans* infection. The protective properties of dsDNA are related to an increased number of CD4^+^ T cells secreting IFN-γ (Remichkova et al., 2009).

One attempt to develop a mucosal vaccination was by using an aspartyl proteinase (Sap2), a very well known enzyme belonging to a family of virulence factors of *C. albicans* (Naglik et al., 2003). Previous clinical and experimental work strongly suggested that Sap2 and possibly other Saps were involved in vaginal infection (Cassone et al., 1987; De Bernardis et al., 1990). Mice immunized with Sap2 showed significantly reduced fungal burdens both orally and vaginally, and rats receiving intravaginal administration of anti-Sap2 antibodies were protected by an intravaginal challenge by *C. albicans*. These studies demonstrated that Sap2 is an immunogenic antigen capable of inducing protective responses against *C. albicans* colonization and infection, and tentatively supports its targeting as a potential vaccine candidate (Rahman et al., 2007). The role of Sap2 in inducing protection against mucosal candidiasis has more recently been underscored by Sandini et al. (2011). These authors generated a recombinant truncated Sap2 protein (rSap2t) and reported that intravaginal immunization with this antigen and cholera toxin as an adjuvant protected from the challenge of a highly vaginopathic strain of *C. albicans*. Protection was possibly due to the elicitation of specific antibodies IgM and IgG anti-rSap2t. More recently rSap2t was incorporated into influenza virosomes, an adjuvant/carrier formulation already used in other human vaccines, which avoids the necessity for a toxin adjuvant. This formulation generated a potent serum antibody response in the mouse and rat following intramuscular immunization. In a rat model of candidal vaginitis the intravaginal or intramuscular administration of rSap2t induced production of anti-Sap2 IgG and IgA in the vaginal fluid, which conferred a consistent degree of protection against vaginal *C. albicans* infection (De Bernardis et al., 2012).

In another study, murine mAb (KT4, IgG1) was used, neutralizing *in vitro* the anti-*Candida* activity as an Id vaccine to elicit Abs. An effective protection that correlated with a significant decrease in vaginal *Candida* CFU was obtained in Id-vaccinated animals compared with controls. The protection was associated with rising vaginal titers of anti-idiotypic Abs (IdAb), prevalently of the IgA isotype, that were able to passively transfer the protective state to non-immunized animals (Polonelli et al., 1994). Since the receptor of the killer toxin recognized by the mAbKT4 is a beta-glucan molecule, it is possible that the protection conferred by the Id vaccine somewhat parallels the protection conferred by the β-glucan-conjugate vaccine (see below).

In a further work, immunization was performed with *C. albicans* heat shock protein 90 kDa (hsp90-CA). Intradermal priming with recombinant hsp90-CA protein, followed by an intranasal or intradermal booster with recombinant hsp90-CA protein, induced significant increases of specific IgG and IgA antibodies in both serum and vaginal fluid. The specific IgG isotype increased after vaginal *Candida* infection, suggesting that *Candida* has the ability to induce a local hsp90-specific antibody (IgG) response during VVC (Raska et al., 2008).

A vaccine composed of β-glucan has been considered a candidate against *Candida* and other fungi. A non-fungal source of β-glucan, laminarin, has been used in these studies. Because polysaccharides are poor immunogens, laminarin was conjugated with the diphtheria toxoid CRM197. This novel glyco-conjugate vaccine administered with human-compatible adjuvant resulted immunogenic and protective as a prophylactic against experimental systemic and mucosal infections by *C. albicans* (Torosantucci et al., 2005). The protection has been ascribed to the production of antibodies to β-(1,3)-glucan (Bromuro et al., 2010). The protective capacity of this β-glucan-conjugate vaccine formulated with the human-compatible MF59 adjuvant was assessed in a murine model of vaginal candidiasis exploiting an *in vivo* imaging technique to monitor the infection. The vaccine conferred significant protection, which was associated to anti-β-glucan IgG antibodies in the serum and in the vagina. The efficacy of the antibodies was demonstrated by the passive transfer of the immune vaginal fluid or anti-β-glucan monoclonal antibodies to naïve mice before infection (Pietrella et al., 2010). The main vaccine candidates are reported in Table 1.

**Table 1:** **Vaccine candidates for protection against candidiasis**.

Components	Reference	Protective immunity	Protection
1,3-β-glucan	Torosantucci et al. (2009), Cassone et al. (2010), Bromuro et al. (2010)	Abs	Systemic
rHyr1p-N	Luo et al. (2011)	Abs	Systemic
Mannoproteins	De Bernardis et al. (2010)	B cells	Vaginal
	Pietrella et al. (2002)	Th1	Systemic
β-1,2-mannotriose-Fba	Xin et al. (2012)	Abs	Systemic
Heat shock proteins 90	Raska et al. (2008)	Abs	Systemic and vaginal
*C. albicans* surface protein, Als3p	Spellberg et al. (2008)	Th1–Th17	Systemic
(Agglutinin-Like Sequence 3) rAls3-N	Baquir et al. (2010), Liu and Filler (2011)	Abs	Vaginal, systemic, and oral
Phosphoglycerate kinase	Calcedo et al. (2012)	Abs	Oral
Secreted aspartic proteases 2	De Bernardis et al. (2012), Cassone and Casadevall (2012)	Abs	Mucosal and vaginal
Yeast derived-β glucan particles (GPs)	Huang et al. (2010)	Th1, Th17, Abs	NT
DCs transfected with fungal RNA	Bozza et al. (2004), Perruccio et al. (2004)	T cell response Th1	Systemic
Nanoparticle-mediated target DCs	Roy and Klein (2012)	T cell response	NT
Liposome-mannan (L-mann)	Han et al. (2000)	Abs to β-1,2-mannotriose	Systemic and vaginal
Daucosterol	Lim et al. (2007)	Th1	Systemic
Enolase	Li et al. (2011)	Abs Th1	Systemic
Glycopeptide or a peptide synthetic vaccine, (*Candida albicans* cell wall-derived)	Xin et al. (2012)	Abs	Systemic
Fba, (peptide derived from fructose bisphosphate aldolase which has cytosolic and cell wall distributions in the fungus)	Cutler et al. (2011)	Abs	Systemic
*C. albicans* dsDNA	Remichkova et al. (2009)	T cells (Th1)	Gastrointestinal

*NT, not tested*.

## Conclusion

Despite several promising approaches, the achievement of a vaccine against mucosal infections in humans still faces a number of problems and challenges. The major challenge remains the difficulties in translating results from rodents to humans, as rodent models can represent at best only part of the variegate patterns of vaginal *Candida* infections in women (Sobel, 2007). In fact most of the data indicating that protection against vaginal infection has been obtained in the rat model, and is not considered by some to be fully representative of the human infection (Fidel and Cutler, 2011). In addition, very few vaccination studies have addressed oral and other types of mucosal candidiasis. Another issue is that vaccine-induced protection must occur in specific niches where *C. albicans* is tolerated as a commensal organism, and it cannot be excluded that affecting fungus colonization by a vaccine could induce some harm in the host by affecting the mucosal microbiome. Other issues concern the search for new vaccine immunogens and adjuvants by existing advanced technologies. It should be taken into account that the most studied vaccines, particularly those in clinical trials (the virosomal Sap2 and the Als3 peptide) have been generated by classical “empiric” approaches, including clinical impressions. Now, a large variety of covalently attached proteins has been found in fungal walls and these proteins can rapidly change their expression in response to different environmental conditions. The knowledge of the specific proteome of the fungus in the host is fundamental for the identification of new vaccine candidates (Klis et al., 2011). Not only proteomics but also complementary post-genomic tools such as transcriptomics and metabolomics in a systems biology context will be useful tools to study pathogen-host interaction, to identify the protective response during infection and the immune response after vaccination. In a recent study, through an integrated analysis of genome-wide transcriptome, Lindqvist et al. determined which signature pathways, processes, and networks are shared by or are exclusive to two classes of experimental vaginal adjuvants, the TLR-9 agonist CpG-ODN and alpha-galactosylceramide in the mouse vagina. These molecular signatures could be used to develop potent mucosal adjuvants that can be used in the female genital tract of a mammal (Lindqvist et al., 2011).

Knowledge of the host mechanisms involved in candidiasis protection, and understanding of the fungal virulence factors, will allow the development of a novel topical mucosal vaccine.

## Conflict of Interest Statement

The authors declare that the research was conducted in the absence of any commercial or financial relationships that could be construed as a potential conflict of interest.
